# Patients undergoing shoulder arthroplasty for failed nonoperative treatment of proximal humerus fracture have low implant survival and low patient-reported outcomes: 837 cases from the Danish Shoulder Arthroplasty Registry

**DOI:** 10.1080/17453674.2020.1730660

**Published:** 2020-02-26

**Authors:** Inger Mechlenburg, Sigrid Rasmussen, Ditte Unbehaun, Alexander Amundsen, Jeppe Vejlgaard Rasmussen

**Affiliations:** a Department of Orthopaedic Surgery, Aarhus University Hospital;; b Department of Clinical Medicine, Aarhus University;; c Department of Public Health, Aarhus University;; d Department of Orthopaedic Surgery, Herlev University Hospital, Denmark

## Abstract

Background and purpose — When nonoperative treatment of proximal humerus fracture (PHF) fails, shoulder arthroplasty may be indicated. We investigated risk factors for revision and evaluated patient-reported outcome 1 year after treatment with either stemmed hemiarthroplasty (SHA) or reverse total shoulder arthroplasty (RTSA) after previous nonoperative treatment of PHF sequelae.

Patients and methods — Data were derived from the Danish Shoulder Arthroplasty Registry and included 837 shoulder arthroplasties performed for PHF sequelae between 2006 and 2015. Type of arthroplasty, sex, age, and surgery period were investigated as risk factors. The Western Ontario Osteoarthritis of the Shoulder index (WOOS) was used to evaluate patient-reported outcome (0–100, 0 indicates worst outcome). Cox regression and linear regression models were used in the statistical analyses.

Results — 644 patients undergoing SHA and 127 patients undergoing RTSA were included. During a mean follow-up of 3.7 years, 48 (7%) SHA and 14 (11%) RTSA were revised. Men undergoing RTSA had a higher revision rate than men undergoing SHA (hazard ratio [HR] 6, 95% confidence interval [CI] 2–19). 454 (62%) patients returned a complete WOOS questionnaire. The mean WOOS score was 53 for SHA and 53 for RTSA. Patients who were 65 years or older had a better WOOS score than younger patients (mean difference 7, CI 1–12). Half of patients had WOOS scores below 50.

Interpretation — Shoulder arthroplasty for PHF sequelae was associated with a high risk of revision and a poor patient-reported outcome. Men treated with RTSA had a high risk of revision.

Displaced proximal humerus fractures (PHF) have been treated nonoperatively, with a locking plate osteosynthesis or with a stemmed hemiarthroplasty (SHA) (Launonen et al. 2019) for many years. More recently, randomized controlled trials have reported similar functional outcome between nonoperative treatment, locking plate osteosynthesis, or SHA for Neer 3 and 4-part fractures (Olerud et al. 2011a, b, Boons et al. 2012, Fjalestad and Hole 2014), but with significantly higher risk of complications and reoperations after operative treatment (Handoll and Brorson 2015, Launonen et al. 2015, Rangan et al. 2015, Beks et al. 2018). This relatively new information may lead to a higher number and proportion of nonoperative treatments in the future.

Fracture sequelae after nonoperative or operative treatment of PHF such as malunion, nonunion, humeral head necrosis, degeneration or tear of the rotator cuff, and secondary gleno­humeral osteoarthritis can lead to severe disability with pain, stiffness of the shoulder, and functional impairment (Greiner et al. 2014, Mansat and Bonnevialle 2015, Brorson et al. 2017). The treatment of fracture sequelae is challenging and the functional outcome after surgery is often disappointing (Kristensen et al. 2018). SHA has been used for decades, but the design depends on intact rotator cuff function and the longevity may be short due to glenoid wear. The reverse total shoulder arthroplasty (RTSA) was initially used in patients with cuff tear arthropathy, but the indication has expanded to other diagnoses, including PHF sequelae (Han et al. 2016). The design of the RTSA does not depend on rotator cuff function, although rotation and stability are improved with intact subscapularis and infraspinatus function.

We investigated risk factors for revision and evaluated patient-reported outcome 1 year after treatment with either SHA or RTSA in previous nonoperative treatment of PHF sequelae.

## Patients and methods

Data were derived from the Danish Shoulder Arthroplasty Registry (DSR), established in January 2004 to monitor and improve shoulder arthroplasty surgery. The registry contains information on primary and revision arthroplasties. Reporting to the DSR has been mandatory for all Danish hospitals and private clinics since 2006 (Rasmussen et al. 2012). The surgeon reports data electronically and patient-reported outcomes are collected by mail 12 months (10–14) after surgery using the Western Ontario Osteoarthritis of the Shoulder index (WOOS) (Rasmussen et al. 2012). The completeness of patients registered in the DSR was 93% during the study period (Danish Shoulder Arthroplasty Registry 2017).

PHF sequelae were defined as fractures reported with nonunion, malunion (including fractures reported together with osteoarthritis), or humeral head necrosis. We included all patients with PHF sequelae reported to the DSR from January 1, 2006 to December 31, 2015. Fractures reported together with previous osteosynthesis were excluded.

### Revision

A revision was defined as removal or exchange of any component or the addition of a glenoid component. The revision was linked to the primary procedure with use of the unique civil registration number assigned to all Danish citizens. The civil registration number is also used when information regarding patients who die or emigrate is derived from the Danish Civil Registration System.

### Patient-reported outcome

The WOOS was used as patient-reported outcome. The WOOS contains 19 questions categorized into 4 domains: physical symptoms, sport and work, lifestyle, and emotions. The patient-reported results are indicated on a visual analogue scale ranging from 0 to 100. The total score ranges from 0 to 1,900 (1,900 indicates worst outcome). To simplify the presentation of the patient-reported results, the raw scores were converted into percentages, where 100 is the best. The Danish version of the WOOS has been culturally adapted and validated for patients with glenohumeral osteoarthritis (Rasmussen et al. 2013). In case of revision, death or emigration within 1 year, the WOOS score was registered as missing.

### Statistics

Descriptive statistics were used to report demographic data and follow-up time. The Kaplan–Meier method was used to illustrate the estimated unadjusted survival rates with 95% confidence interval (CI). The Cox regression model was used to determine the hazard ratios (HR) of revision with a CI. Arthroplasty type, age, sex, and surgery period were included in the multivariate model and the linear regression model. We used 2 age categories: younger than 65 years, and 65 years or older. This categorization was applied based on the Danish retirement age, due to an expected change in the patient’s activity level. We used 2 surgery periods, 2006–2010 and 2011–2015. Patient data contributed with individual risk time until revision, emigration, death, or until December 31, 2015, whichever came first. Test of proportional-hazards assumption was considered fulfilled. Although it violates the assumption of independence, patients with bilateral shoulder arthroplasty procedures were included in the survival analysis as if they were independent (Ranstam et al. 2011).

A linear regression model was used to estimate the predicted mean difference in the WOOS score.

Arthroplasty type, age, sex, and surgery period were included in the multivariate model. A plot of residuals versus predicted values, plots of residuals versus independent variables, and a normal probability plot of the residuals were used to check whether the assumptions of linearity, independence, constant variance, and normality of the residuals were fulfilled. Characteristics of patients responding or not responding to the WOOS questionnaire were compared using the chi-square test for categorical variables and Student’s t-test for continuous variables. The level of statistical significance was set at p < 0.05 and all p-tests were 2-tailed. The analyses were performed using STATA 15.0 (StataCorp LP, College Station, TX, USA).

### Ethics, funding, data sharing, and potential conflicts of interest

According to Danish law, ethics committee approval was not required. No funding was obtained for this study. As part of the Data Use Agreement at the Danish Shoulder Arthroplasty Registry, authors are not allowed to provide raw data. Upon reasonable request, the corresponding author will provide statistical programming codes used to generate the results. No potential conflicts of interests are declared.

## Results

### Demographics

**Figure 1.**
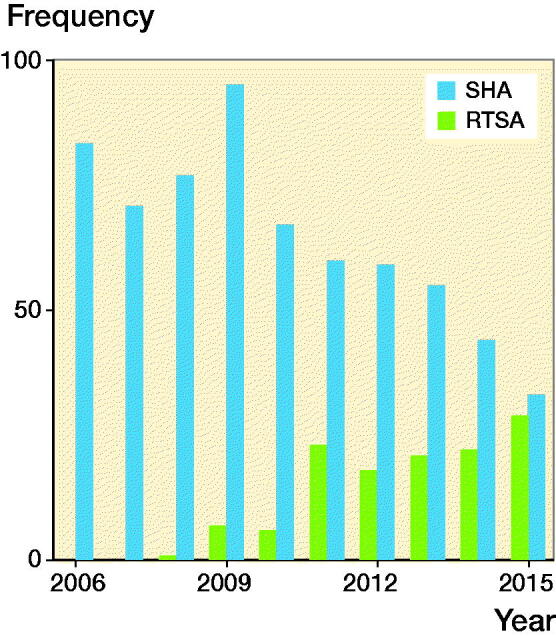
Number of hemiarthroplasties (SHA) and reverse shoulder arthroplasties (RTSA) due to failed nonoperative treatment of proximal humerus fracture registered in the Danish Shoulder Arthroplasty Registry, 2006–2015.

837 patients were treated with shoulder arthroplasty for sequelae after a previous nonoperatively treated PHF; 644 underwent SHA and 127 underwent RTSA (Figure 1). 2% of patients had bilateral shoulder arthroplasty performed. Women accounted for 71% of arthroplasties. Mean age was 70 (SD 11) years, and 69% of patients were older than 65 years. The reasons for sequelae were nonunion (67%), malunion (27%), or humeral head necrosis (7%) (Table 1).

**Table 1.** Demographics of the study population presented by other type of arthroplasties (Others), stemmed hemiarthroplasty (SHA), and reverse shoulder arthroplasty (RTSA). Values are n (%) unless otherwise specified

	Others	SHA	RTSA	Total **^a^**
Factor	(n = 65)	(n = 644)	(n = 127)	(n = 837)
Sex				
Women	44 (68)	462 (72)	89 (70)	595 (71)
Men	21 (32)	182 (28)	38 (30)	242 (29)
Age				
< 65	19 (29)	206 (32)	32 (25)	258 (31)
≥ 65	46 (71)	438 (68)	95 (75)	579 (69)
Age, mean (SD)	69 (11)	70 (11)	71 (9.4)	70 (11)
Indication for surgery				
Malunion ^b^	33 (51)	134 (21)	55 (43)	222 (27)
Nonunion	23 (35)	470 (73)	61 (48)	555 (67)
Caput necrosis	9 (14)	37 (6)	10 (8)	56 (7)
Missing	–	3 (0)	1 (1)	4 (0)
Period of surgery				
2006–2010	25 (38)	393 (61)	14 (11)	432 (52)
2011–2015	40 (62)	251 (39)	113 (89)	404 (48)
Revision	9 (14)	48 (7)	14 (11)	71 (8)
WOOS (completed)	37 (66)	347 (61)	70 (66)	454 (62)
WOOS mean (SD)	38 (26)	47.4 (26)	47.5 (26)	46.6 (26)

**^a^**For 1 shoulder arthroplasty, there was missing information on

the type of arthroplasty with which the patient was treated.

**^b^**Malunion includes fractures reported together with osteoarthritis.

WOOS: Western Ontario Osteoarthritis of the Shoulder index.

### Risk of revision

**Figure 2.**
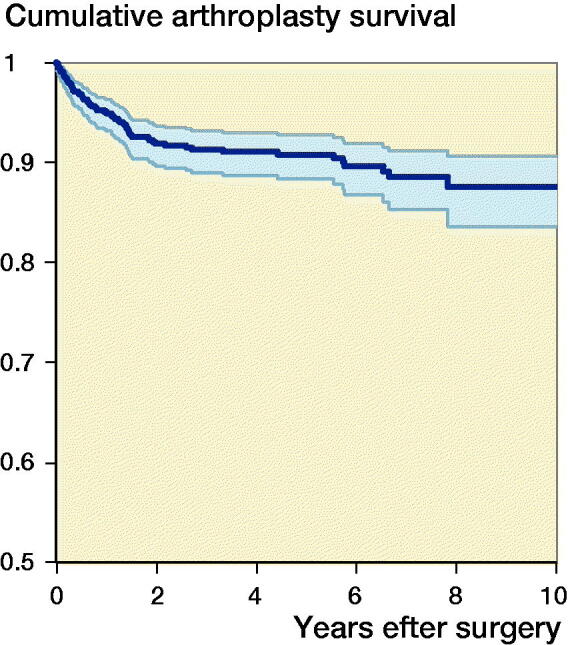
Cumulative survival for all types of arthroplasties, 2006–2015

The median follow-up time was 3.2 years (IQR 1.3–6.1). 71 (8%) of the shoulder arthroplasties were revised (Figure 2). The 1-, 5-, and 10-year cumulative arthroplasty survival rates with CI for women were 97% (95–98), 92% (89–94), and 91% (87–93) for SHA, and 94% (86–98) and 92% (84–97)for RTSA. For men 1-, 5-, and 10-year cumulative survival rates with CI were 94% (92–98), 92% (87–96), and 82% (65–92) for SHA, and 80% (62–90) and 76% (57–87) for RTSA, respectively (Figure 3). There were no statistically significant differences in risk of revision among arthroplasty type, age, sex, or surgery period (Table 2). However, sex had a significant impact on the result of arthroplasty. Men treated with RTSA had a higher risk of revision than men treated with SHA (HR 6.0, CI 1.9–19) (Table 3). The cumulative survival rates for men were significantly higher for SHA compared with RTSA (Figure 3). Overall, the most common reasons for revision were dislocation (28%), rotator cuff problems (17%), other reasons (includes pain with no other reasons reported or malposition) (16%), and infection (11%) (Table 4).

Figure 3.Cumulative survival for SHA (blue) and RTSA (red) in women (upper panel) and men (lower panel).

**Figure:**
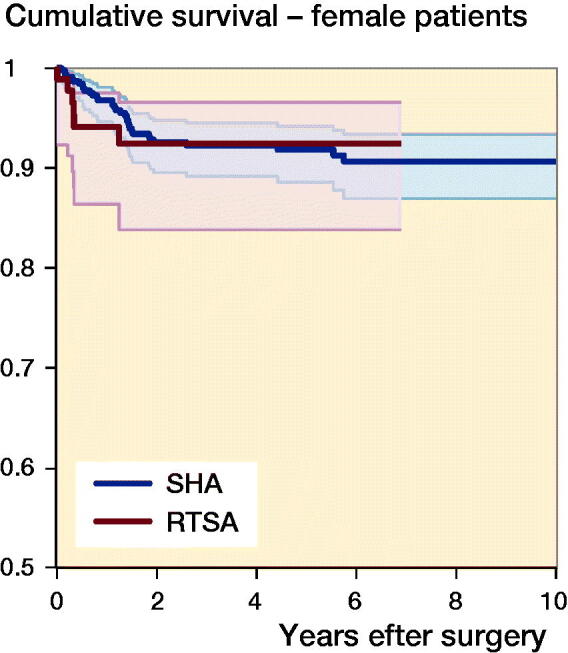

**Figure:**
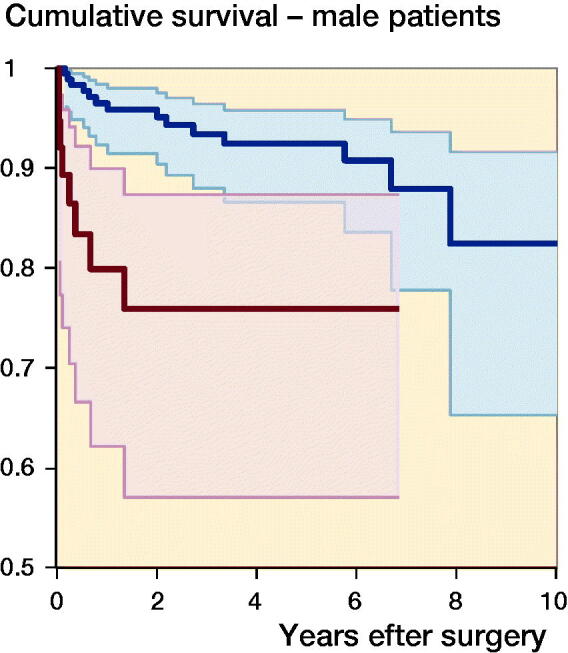

**Table 2.** Univariate and multivariate Cox regression model for revision of stemmed hemiarthroplasty (SHA), reverse shoulder arthroplasty (RTSA), sex, age, and period of surgery, (n = 771, revisions = 62)

	Univariate	Multivariate			
Factor	HR (CI)	p-value	HR (CI)	p-value	
Type of arthroplasty					
SHA	1		1		
RTSA	2.0 (1.1–3.7)	0.02	1.1 (0.4–2.7)	0.8	
Sex					
Women	1		1		
Men	1.4 (0.8–2.4)	0.2	1.0 (0.5–1.9)	1.0	
Age					
< 65	1		1		
≥ 65	0.7 (0.4–1.2)	0.2	0.7 (0.4–1.2)	0.2	
Period of surgery					
2006–2010	1		1		
2011–2015	1.6 (0.9–2.6)	0.1	1.3 (0.7–2.3)	0.4	
SHA + women			1		
RTSA + men			3.5 (1.0–12)	0.05	

HR (CI): Hazard ratio (95% confidence interval).

SHA: stemmed hemiarthroplasty,

RTSA: reverse shoulder arthroplasty.

**Table 3.** Multivariate Cox regression model for revision of women (n = 551, revisions = 40) and men (n = 220, revisions = 22) adjusted for age and period of surgery

	Women	Men		
Type of arthroplasty	HR (CI)	p-value	HR (CI)	p-value
SHA	1		1	
RTSA	1.0. (0.4–2.4)	0.9	6.0 (1.9–19)	0.003

For abbreviations, see Table 2.

**Table 4.** Reasons for revision for all types of arthroplasties (All), other types of arthroplasties (Others), stemmed hemiarthroplasty (SHA), and reverse shoulder arthroplasty (RTSA). Values are n, (percentage of primary arthroplasties), and percentage of revisions

Reasons for revision	All **^a^** (n = 837)		Others (n = 65)		SHA (n = 644)		RTSA (n = 127)	
Dislocation	23 (2.7)	28	3 (5)	27	12 (1.9)	21	8 (6.3)	62
Loosening	3 (0.4)	4	0 (0)	0	2 (0.3)	4	1 (0.8)	8
Glenoid wear	5 (0.6)	6	0 (0)	0	5 (0.8)	9	0 (0)	0
Infection	9 (1.1)	11	2 (3)	18	6 (0.9)	11	1 (0.8)	8
Fracture	5 (0.6)	6	0 (0)	0	5 (0.8)	9	0 (0)	0
Technical failure	8 (1.0)	10	1 (2)	9	7 (1.1)	12	0 (0)	0
Rotator cuff problems	14 (1.7)	17	2 (3)	18	12 (1.9)	21	0 (0)	0
Other reasons ^b^	13 (1.6)	16	2 (3)	18	8 (1.2)	14	3 (2.4)	23
Missing	1 (0.1)	1	1 (2)	9	0 (0)	0	0 (0)	0
Total	81 (9.7)	100	11 (17)	100	57 (8.9)	100	13 (10.2)	100

**^a^** For 1 shoulder arthroplasty, there was missing information on the type of arthroplasty with which the patient was treated.

**^b^**Other reasons includes pain with no other reasons reported.

### Patient-reported outcome

0.5% of the patients emigrated or were foreign citizens. 7% of patients died and 5% of patients underwent revision within the first year after surgery. Thus, the WOOS questionnaire was sent to 732 patients; 62% of patients returned a complete WOOS, 6% returned an incomplete questionnaire, and 32% did not respond.

**Figure 4.**
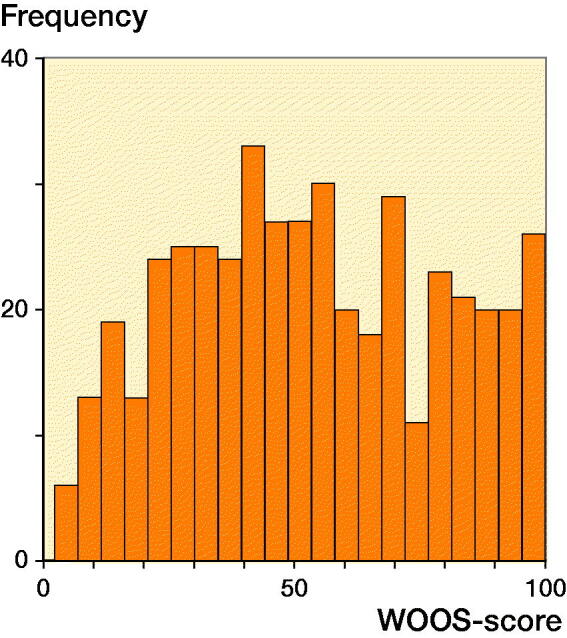
Distribution of WOOS scores at the 1-year follow-up for all patients.

1 year after shoulder arthroplasty, the mean WOOS score was 53 (SD 26) for the entire cohort, 53 (SD 26) for patients treated with SHA and 53 (SD 26) for patients treated with RTSA (Figure 4). 49% of the patients had a WOOS score below 50. There was no significant difference between the WOOS score of SHA and RTSA in the multivariate regression model (mean difference = –1.9, CI –9.2 to 5.5) (Table 5.) Patients 65 years or older had a better WOOS score compared with younger patients (mean difference = 6.6, CI 1.1–12), but the differences were not considered clinically relevant (Table 5). There was no statistically significant difference in type of arthroplasty, sex, age, and surgery period between patients who responded or did not respond to the WOOS (Table 6); this indicates that the patients responding to the WOOS in this study were not selected.

**Table 5.** Univariate and multivariate linear regression model for mean difference (MD) in Western Ontario Osteoarthritis of the Shoulder score, type of arthroplasty, sex, age, and period of surgery, (n = 417)

	Univariate	Multivariate		
Factor	MD (CI)	p-value	MD (CI)	p-value
Type of arthroplasty				
SHA	0		0	
RTSA	–0.1 (–6.8 to 6.5)	1.0	–1.9 (–9.2 to 5.5)	0.6
Sex				
Women	0		0	
Men	1.7 (–3.9 to 7.4)	0.6	3.5 (–2.3 to 9.3)	0.2
Age				
< 65	0		0	
≥ 65	5.8 (0.5 to 11)	0.03	6.6 (1.1 to 12)	0.02
Period of surgery				
2006–2010	0		0	
2011–2015	2.1 (–3.0 to 7.1)	0.4	2.4 (–3.1 to 8.0)	0.4

**Table 6.** Comparison of patients responding or not responding to the Western Ontario Osteoarthritis of the Shoulder index (WOOS). Values are n (%) unless otherwise specified

	Responders	Non-responders	
Factor	(n = 454)	(n = 278)	p-value
Type of arthroplasty			0.5
SHA	347 (76)	222 (80)	
RTSA	70 (15)	36 (13)	
Others	37 (8)	19 (7)	
Sex			0.7
Women	329 (72)	198 (71)	
Men	125 (28)	80 (29)	
Age			0.7
< 65	145 (32)	85 (31)	
≥ 65	309 (68)	193 (69)	
Age mean (SD)	69.4 (11.0)	70.1 (11.4)	0.1
Indication for surgery **^a^**			0.4
Malunion ^b^	128 (28)	66 (24)	
Nonunion	293 (65)	191 (69)	
Caput necrosis	32 (7)	20 (7)	
Period of surgery			0.2
2006–2008	146 (32)	76 (27)	
2009–2011	139 (31)	100 (36)	
2012–2015	169 (37)	102 (37)	

**^a^** Responders, n = 453. Non-responders, n = 277, stemmed hemi-arthroplasty (SHA), and reverse shoulder arthroplasty (RTSA).

**^b^** Malunion includes fractures reported together with osteoarthritis.

## Discussion

The main findings in this study were the poor patient-reported outcomes and the low implant survival rate, especially for men undergoing RTSA.

We found a 5-year cumulative survival rate of 76% for men undergoing RTSA, which is less than we would usually accept, particularly because revision of RTSA is complex and challenging due to the design of the RTSA and limited glenoid bone stock (Brorson et al. 2017, Holton et al. 2017). In our study, dislocation was the indication for revision in two-thirds of patients with revised RTSA. The reason for dislocations cannot be deduced from this registry study. It may be related to difficulties in placing the humeral component appropriately with correct tensioning of the deltoid muscle due to changed bone morphology. It may also be related to malposition or reabsorption of the tubercles and thus the function of the infraspinatus and subscapularis muscles. This could add imbalance to the reversed design. The survival rate of SHA seems more promising, but it is difficult to compare the 2 arthroplasty types directly. The RTSA may have been used for the most severe cases and in patients with a rotator cuff problem. Thus, the survival rate of SHA would probably not be as good if it had been used in the same patients. Our findings indicate that RTSA is not the easy solution for patients with PHFS and based on the high risk of revision in general and dislocation in particular we suggest that RTSA for PHFS is performed by experienced surgeons only and that surgeons who perform the operation focus their attention on technical details.

Information concerning patient-reported outcome after shoulder arthroplasty for failed, nonoperative treatment of PHF is sparse. A retrospective study reported the mean Constant score in 42 patients being operated with a RTSA due to malunion of a PHF and found that the Constant score increased from 20 points (0–52 points) preoperatively to 55 points (21–83 points) 4 years postoperatively (Raiss et al. 2016). The complication rate was 10%. Raiss et al. also reported the Constant score in 32 patients operated with an RTSA due to nonunion of a PHF and found that the Constant score increased from 14 points (2–35 points) to 47 points (6–75 points) 4 years postoperatively (Raiss et al. 2014). There were, however, complications in 41% of patients leading to revision of 28% of the arthroplasties. The Constant score and the WOOS are not directly comparable but the results from these studies seem similar to our WOOS score of 53.

Kristensen et al. (2018) evaluated patient-reported outcome in patients with PHF initially treated with osteosynthesis and later treated with shoulder arthroplasty. The authors used data from the DSR and found a WOOS score of 46 (SD 25). As in our study there was no difference in WOOS between SHA and RTSA. Thus, based on data from DSR there seems to be no difference in WOOS between patients previously treated nonoperatively and patients who have had previous osteosynthesis.

We found that patients aged above 65 years achieved a significantly better WOOS score compared with younger patients. However, the difference was small and may not be clinically relevant. Poor patient-reported outcomes have also been reported for younger patients with osteoarthritis (Rasmussen et al. 2014). The reason is unknown and cannot be deduced from this registry study, but it may be related to higher expectations and higher functional demands. Overall, the patients achieved poor patient-reported outcomes 1 year after shoulder arthroplasty and 49% of patients reported WOOS scores < 50, interpreted as a clinical failure.

### Methodological considerations

Although there was no difference in type of arthroplasty, sex, age, and surgery period between patients who responded or who did not respond to the WOOS, the relatively low number of patients responding to the WOOS may have affected the results. The WOOS was developed and validated for patients with osteoarthritis and the validity of applying WOOS as an outcome for patients with PHF sequelae has not been investigated. Although the WOOS is used in the Danish and Swedish shoulder registries and from 2020 introduced in the Finnish and Norwegian shoulder registries, the WOOS is not widely used, which makes comparison with results from other countries difficult. Although the correlation between the WOOS and the Constant score and the Oxford Shoulder Score is high (Rasmussen et al. 2012), direct comparability is not possible. The size of the study population and the number of events was relatively low, especially since the regression models included several variables. Furthermore, the follow-up time was short and loosening will usually not appear within 1 year postoperatively. The indications for undergoing SHA and RTSA might have been different, introducing selection bias. In addition, there is risk of wrong coding when registering to the DSR. Moreover, unknown confounders may have influenced the results. The literature reports that comorbidities, smoking, body mass index, drug and alcohol abuse are frequent in patients with fracture sequelae and that these factors affected the outcome of shoulder arthroplasty (Murray et al. 2011, Werner et al. 2015, Hernandez et al. 2017). Unfortunately, the DSR does not collect this type of information. Neither does the DSR collect information on preoperative shoulder pain and function. Finally, not all patients are treated with arthroplasty after PHF sequelae and there is most likely a selection bias towards more healthy, active, and demanding patients. An unknown proportion of patients will not be offered or will not accept shoulder arthroplasty, even if they have a poor outcome. Hence, it is a strength of our study that revision rates are supplemented with patient-reported outcome, which shows that half of patients reported high levels of pain 1 year after shoulder arthroplasty.

In conclusion, we found low survival rates in patients undergoing SHA and RTSA for PHF sequelae. In particular, men undergoing RTSA had a high risk of revision. The patient-reported outcomes were poor with half of patients reporting WOOS score below 50 with no difference between SHA and RTSA or between men and women. The findings indicate that RTSA is not the easy solution for PHF sequelae. It is a technically demanding operation and we suggest that caution is warranted when considering treating PHF sequelae with RTSA in men.
